# Secondary Involvement of the Mandible due to Basal Cell Carcinoma: A Case Report

**Published:** 2015-05

**Authors:** Pegah Mosannen Mozaffary, Zahra Delavarian, Maryam Amirchaghmaghi, Zohreh Dalirsani, Leila Vazifeh Mostaan, Shadi Saghafi Khadem, Hanieh Ghalavani

**Affiliations:** 1 Oral and Maxillofacial Diseases Research Center, Department of Oral Medicine, School of Dentistry, Mashhad University of Medical Sciences, Mashhad, Iran;; 2 Dental Research Center, Department of Oral Medicine, School of Dentistry, Mashhad University of Medical Sciences, Mashhad, Iran;; 3 Cancer Research Center, Omid Hospital, Faculty of Medicine, Mashhad University of Medical Sciences, Mashhad, Iran;; 4 Oral and Maxillofacial Diseases Research Center, Department of Oral and Maxillofacial Pathology, School of Dentistry, Mashhad University of Medical Sciences, Mashhad, Iran;; 5 Oral Medicine Specialist, Mashhad, Iran

**Keywords:** Basal cell carcinoma, Intraoral, Cancer

## Abstract

Basal cell carcinoma (BCC) is the most common cutaneous malignancy among Caucasians. Rare examples of aggressive and neglected BCC have been reported. Here we report a unique case of a neglected BCC with significant jaw involvement.

A 50-year-old female, referred by an otorhinologist, presented with a large ulcer on her chin, which was extended to her mandibular vestibule. The ulcer was 9×5.5 cm in size, and tissue destruction, necrosis was observed in the central portion, and the mandibular bone was exposed. On intraoral examination, tooth mobility and severe bone loss were evident. Due to the primary cutaneous origin of the lesion, BCC was considered as preliminary diagnosis. Biopsy was performed and diagnosis of BCC was confirmed. The diseased mandibular bone was resected and reconstructed with a surgical plate. The soft tissue defect was reconstructed with deltopectoral flap. The patient refused secondary stage plastic surgery.

Although BCC is not a lethal malignancy, if left untreated and neglected, it can result in severe destruction, disfigurement, and even mortality.

## Introduction

Basal Cell Carcinoma (BCC) is the most common type of skin cancer, particularly among Caucasians, and 85% of BCC are located in the head and neck area.^1^ It occurs primarily in fair-skinned people who work or spend a considerable amount of daylight time outdoors being exposed to ultraviolet derived from the sun.^2-4^ The main causes of BCC are ultraviolet (UV) light, industrial chemical substances such as vinyl chloride, polycyclin, hydroxycarbamide , and alkalizing agents.^5^^,^^6^

It occurs more often in men than in women, perhaps due to the use of cosmetics tanning coatings and light sunbeds by women.^2^ It is seen more often after the age of 50, but in patients younger than 35 it is more aggressive.^1^

BCC is mainly located on sun-exposed sites; being the head and neck (partially in upper face), are the areas of more incidences.^2^^,^^7^ Patients with BCC are at increased risk of experiencing squamous cell carcinoma (SCC) and malignant melanoma.^2^^,^^8^ The risk of BCC has been demonstrated in patient who have received radiation or immunosuppressive treatment.^2^ Other risk factors include blond hair, blue or green eyes, freckles, etc. The nevoid BCC syndrome or Gorlin‘s syndrome is a condition with significant oral, head and neck involvement that occurs at a younger age (under 30 years) and should be recognized by dentists. The syndrome consists of multiple BCCs, odontogenic keratocysts of jaws, palmer, or planter pits, anomalies of the ribs and spine (bifid ribs, spina bifida) and calcification of the falx cerebri.^2^^,^^8^ Here we report a unique case of a neglected BCC with significant jaw involvement. The patient underwent mandibulectomy and reconstructive surgery was performed.

## Case Report

**Figure 1:**
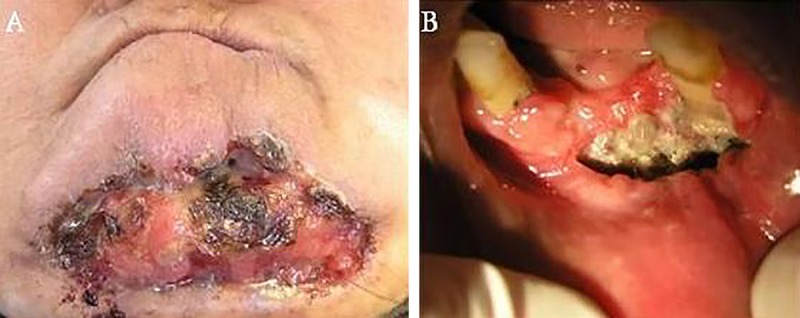
A) Large ulcer with firm and indurated margins on the chin skin was observed in clinical examination. B) The ulcer has extended to the mandibular vestibule (see the necrosis in the surrounding bone).

**Figure 2:**
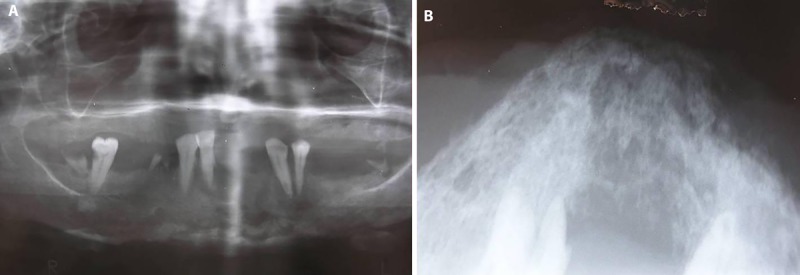
A) An ill-defined radiolucency of the mandibular symphysis in panoramic view. B) Due to super imposition of neck vertebrae an occlusal view was ordered. The ill-defined radiolucency of the mandibular symphysis is observed.

**Figure 3:**
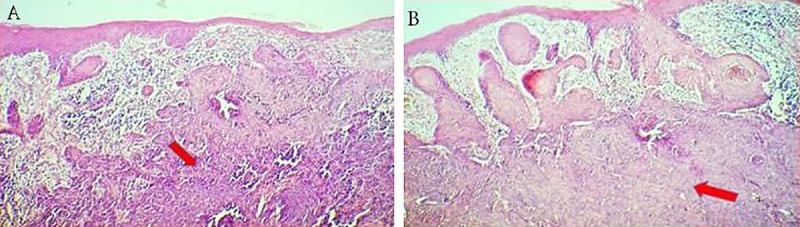
A) Uniform ovoid and dark staining basaloid cells with moderate sized nuclei and relatively little cytoplasm are observed. B) Basaloid cells are arranged into well-demarcated islands and strands, which appeared to be raised from the basal cell layer of the overlying epidermis.

**Figure 4:**
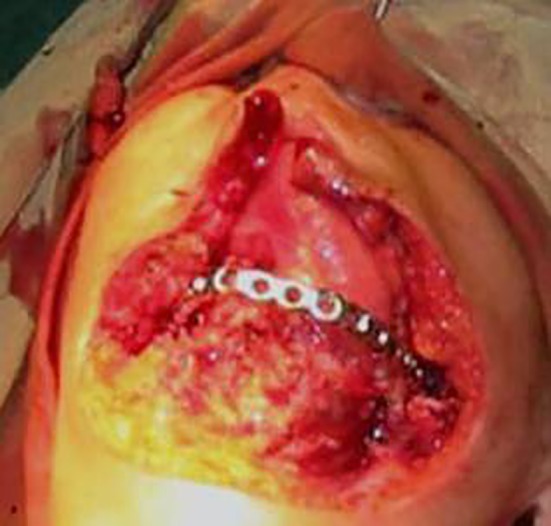
The diseased muscle and mandibular bone are resected and a surgical plate is used for reconstruction.

**Figure 5:**
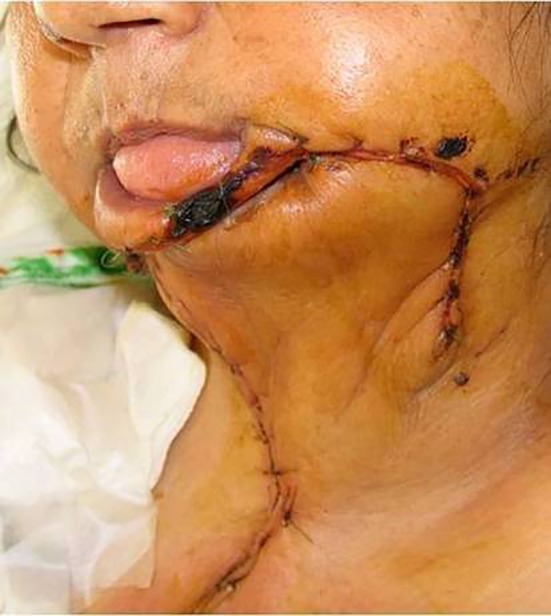
The soft tissue defect is reconstructed with deltopectoral flap. The patient refused second stage plastic surgery. The final outcome is an acceptable reconstruction, but the lips remained incompetent.

A 50-year-old female was referred by an otorhinologist with a large chin ulcer and toothache. The medical history was unremarkable, having never had a medical consultation due to the poor socioeconomic situation. There was a 20-year history of gradually enlarging black papule on her chin. The patient complained of unpleasant facial appearance and a dull pain in the chin region. On extra oral examination, there was a large ulcer (10×6.5 cm) with a firm and indurated margins on the chin extending to the mandibular vestibule and necrosis was evident in surrounding bone (figure 1). On palpation, drainage of pus was evident from the ulcer. The mandibular right lateral incisor and canines were mobile. Central incisors were prior to admission due to extreme mobility. Radiographic examination revealed an ill-defined radiolucency of the mandibular symphyses (figure 2). Cytological examination did not show any evidence of spirochete infection. Although the lesion had an atypical appearance, the slow course, in addition to clinical characteristics such an enrolled boarders, destructive ulcer, and concurrent involvement of skin and bone led the diagnosis of BCC as the first possibility. Incisional biopsy was performed at the margin of the ulcer. Other differential diagnosis included necrotizing ulcerative gingivostomatitis with facial involvement (noma), osteomyelitis, leishmaniasis, Wegener granulomatosis, deep fungal infection, and amelanotic melanoma. The histopathologic sample consisted of uniform ovoid, and dark staining basaloid cells with moderate sized nuclei and relatively little cytoplasm. The cells were arranged into well-demarcated islands and strands, which appeared to be raised from the basal cell layer of the overlying epidermis and invaded into the underlying connective tissue (figure 3). The histopathologic evaluation of the resected bone showed secondary osteomyelitis due to the extensive ulcer. The clinical diagnosis of BCC was confirmed. Surgical resection was planned for the lesion. During the surgery, the involved muscle and mandibular bone were resected and reconstructed with a total mandibular reconstruction plate (figure 4). The soft tissue defect was reconstructed with deltopectoral flap. The patient refused second stage plastic surgery, however, the final outcome was an acceptable reconstruction, but the lips remained incompetent (figure 5). The patient was followed-up for 3 years and no recurrence was observed. A written consent was obtained from the patient for medical case report.

## Discussion

Most BCCs involve the head and neck region (80%) and the reminders involve the trunk and limbs.^5^ Here we report a unique neglected case of BCC with extensive mandibular and intraoral involvement. At the first visit, one might consider lesions with mandibular origin, but further examination and accurate history taking led to the diagnosis of BCC. BCC has a gradual growth without any pain or complaint, which may lead to negligence. Occasionally, BCC behaves aggressively and invades deep structures, recurs after treatment and metastasizes to regional and distant lymph nodes.^5^ BCC has several types due to clinical and histopathological characteristics as nodular, superficial, micronodular, morphemic form, infiltrating, pigmented, metatypic and fibroepithelioma of pinkus.^3^ Nodular variant is the most frequent form of BCC and, if left untreated, may result in a large eroding tumor with central depression, necrosis of underlying tissue and raised and rolled borders.^2^^,^^4^ There are several reports about aggressive and neglected BCC of the head and neck with terrible clinical outcomes. Asillian et al. reported on a 58-year-old man with an extensive BCC and signs of cranial nerve involvement with a large, infected ulcer (15×15 cm) on his scalp. The patient had skull bone destruction, osteomyelitis, mastoiditis, cranial nerve paralysis, and radiographic features of the skull base and upper cervical soft tissue involvement. Pathological studies revealed an infiltrating form of BCC. The patient provided a history of receiving an unknown amount of radiation to the scalp for the treatment of tinea capitis during childhood. The patient died because of cranial involvement.^9^ Rishiraj et al. reported on three cases of BCC with maxillofacial involvement. One case had extensive ulcer affecting right facial structures such as antrum, orbit, and hard palate. A purulent discharge and necrosis was evident. The patient refused any treatment. Other cases were treated by radiotherapy.^3^^,^^9^

Sebastian reported on a 77-year-old with infiltrating ulcer from the eyelid to ocular globe. The biopsy confirmed BCC. Cranial tomography showed destruction of the orbital inferior wall and infiltration from papyraceous lamina to the ipsilateral maxillary antrum. A superstructure maxillectomy with left orbital exertion was performed. Eight months after the procedure, the patient remained without symptoms of tumoral activity.^7^ Hatano et al. reported on two cases of BCC that developed and invaded the preorbital and lacrimal region.^8^ Nagler and Laufer reported on three patients with BCC lesions invading their jaws, as a consequence of which either their mandible or maxilla had to be partially resected.^10^

There are many treated modalities for BCC such as surgical treatment, microscopic controlled surgery (Mohs surgery), electric cauterization and curettage, cryotherapy, roentgen therapy, laser treatment, 5-flourouracil, imiquimod, interferon alpha, and photodynamic therapy.^5^^,^^6^ The goal of BCC treatment is complete excision of the tumor with the preservation of surrounding structures and careful follow-up to detect recurrence or new primary tumors if BCC is operable.^9^ In some areas such as orbit, nose, and external ear, surgery is complicated and other forms of treatment must be considered. The rate of recurrence is about 1% with Mohs surgery and up to 10% for other forms of treatment. Smaller basal cell carcinomas are less likely to recur than larger ones. BCC rarely spread to other parts of the body, but if involves vital structures such as calvarium, it can be fatal. Our case presented with a destructive ulcer in the chin with mandibular involvement. Preliminary diagnosis was BCC due to the prolonged clinical course, enrolled borders, and patient’s negligence. Differential diagnosis consisted of necrotizing ulcerative gingivitis with facial involvement (noma), leishmaniasis, Wegener granulomatosis, deep fungal infection, and amelanotic melanoma. Amelanotic melanoma was ruled out because of a long clinical course of the lesion. Leishmaniasis cannot cause bone necrosis and has a moderate clinical course. Noma was excluded, because the lesion was initiated from the skin and the patient reported bone involvement as a secondary complaint. In addition, cytological evaluation did not reveal spirochetes. The absence of respiratory symptoms and the location of the lesion, ruled out deep fungal infection and Wegener granulomatosis. The preliminary diagnosis was confirmed by biopsy and the patient underwent surgery. No other treatment such as radiotherapy and chemotherapy was necessary. The patient refused second stage plastic surgery. The final outcome was an acceptable reconstruction, but the lips remained incompetent.

## Conclusion

BCC is a dermatologic disease, with a high prevalence in the head and neck but rarely can involve maxillofacial bones and oral cavity. Therefore, dentists should be familiar with this disease and possible unpleasant outcomes. 
